# Early detection of cognitive decline in Alzheimer’s disease using eye tracking

**DOI:** 10.3389/fnagi.2023.1123456

**Published:** 2023-03-21

**Authors:** Shin-ichi Tokushige, Hideyuki Matsumoto, Shun-ichi Matsuda, Satomi Inomata-Terada, Naoki Kotsuki, Masashi Hamada, Shoji Tsuji, Yoshikazu Ugawa, Yasuo Terao

**Affiliations:** ^1^Department of Neurology, Graduate School of Medicine, The University of Tokyo, Tokyo, Japan; ^2^Department of Neurology, Kyorin University, Tokyo, Japan; ^3^Department of Neurology, Mitsui Memorial Hospital, Tokyo, Japan; ^4^Department of Neurology, NTT Medical Center Tokyo, Tokyo, Japan; ^5^Department of Medical Physiology, Kyorin University, Tokyo, Japan; ^6^Institute of Medical Genomics, International University of Health and Welfare, Chiba, Japan; ^7^Department of Human Neurophysiology, Fukushima Medical University, Fukushima, Japan

**Keywords:** Alzheimer’s disease, cognitive decline, eye tracking, fixation, saccade, pupil

## Abstract

**Background:**

Patients with Alzheimer’s disease (AD) are known to exhibit visuospatial processing impairment, as reflected in eye movements from the early stages of the disease. We investigated whether the pattern of gaze exploration during visual tasks could be useful for detecting cognitive decline at the earliest stage.

**Methods:**

Sixteen AD patients (age: 79.1 ± 7.9 years, Mini Mental State Examination [MMSE] score: 17.7 ± 5.3, mean ± standard deviation) and 16 control subjects (age: 79.4 ± 4.6, MMSE score: 26.9 ± 2.4) participated. In the visual memory task, subjects memorized presented line drawings for later recall. In the visual search tasks, they searched for a target Landolt ring of specific orientation (serial search task) or color (pop-out task) embedded among arrays of distractors. Using video-oculography, saccade parameters, patterns of gaze exploration, and pupil size change during task performance were recorded and compared between AD and control subjects.

**Results:**

In the visual memory task, the number of informative regions of interest (ROIs) fixated was significantly reduced in AD patients compared to control subjects. In the visual search task, AD patients took a significantly longer time and more saccades to detect the target in the serial but not in pop-out search. In both tasks, there was no significant difference in the saccade frequency and amplitude between groups. On-task pupil modulation during the serial search task was decreased in AD. The number of ROIs fixated in the visual memory task and search time and saccade numbers in the serial search task differentiated both groups of subjects with high sensitivity, whereas saccade parameters of pupil size modulation were effective in confirming normal cognition from cognitive decline with high specificity.

**Discussion:**

Reduced fixation on informative ROIs reflected impaired attentional allocation. Increased search time and saccade numbers in the visual search task indicated inefficient visual processing. Decreased on-task pupil size during visual search suggested decreased pupil modulation with cognitive load in AD patients, reflecting impaired function of the locus coeruleus. When patients perform the combination of these tasks to visualize multiple aspects of visuospatial processing, cognitive decline can be detected at an early stage with high sensitivity and specificity and its progression be evaluated.

## Introduction

In the aging population, there is an ever-increasing number of patients with Alzheimer’s disease (AD), a common form of dementia characterized by progressive memory disorder (Lopez and Kuller, 2019). Along with the deposition of tau protein and amyloid-beta protein, neurodegeneration occurs in multiple brain areas such as the hippocampus, posterior cingulate gyrus, and the associative areas of the cerebral cortex in AD.

The mainstay therapy using acetylcholine esterase inhibitors and NMDA receptor antagonists is largely symptomatic and provides no real cure. New disease-modifying therapy to stop/remove the amyloid accumulation does not reverse the cognitive impairment that has already set in. Given that disease progression becomes irreversible at some stage of neurodegenerative progression (Viña and Sanz-Ros, 2018), early diagnosis is essential in the context of novel treatment strategies. Reversing cognitive decline by restoring the metabolic process at an early stage has emerged as a novel and promising measure for improving cognitive decline in AD and for improving the quality of life in patients (Bredesen, 2014; Bredesen et al., 2016). In this context, the challenge is how early we can make an accurate diagnosis, that is, at the very beginning of cognitive impairment. Conventional neuroimaging such as computed tomography (CT) and magnetic resonance imaging (MRI) can detect brain atrophy only after neurodegeneration has progressed to a certain stage. Amyloid positron emission tomography (PET) and cerebrospinal fluid tau protein may enable earlier diagnosis by detecting the accumulation of amyloid, but these tests are costly, not performed by all facilities, and only available to a limited number of patients. There is an imminent need for developing methods that can diagnose AD at its earliest stage with high sensitivity and reasonable cost.

Based on the recognition that abnormalities of eye movement appear very early in the disease course of AD, many studies have attempted to use eye movement tasks for the detection of cognitive impairment. Elementary oculomotor tasks for saccades have reported slower reaction times than controls in gap and overlap tasks (Crawford et al., 2015), latency correlating with Mini Mental State Examination (MMSE) score (Yang et al., 2013), decreased accuracy of saccades made toward the instructed target contained in presented photographs or figure sets (Boucart et al., 2014; Lenoble et al., 2015), and an increased proportion of (uncorrected directional) errors made by AD patients as compared to control subjects in antisaccade tasks, in which subjects have to make saccades in the opposite direction of the presented target (Crawford et al., 2013; Heuer et al., 2013). Studies have also demonstrated that eye tracking can be used to detect cognitive decline in AD and assess disease progression (Crawford et al., 2015; Beltrán et al., 2018, for review). Some studies have addressed more cognitive aspects of oculomotor behavior, for example, during a reading task, which involves working memory and memory retrieval function. Researchers have reported failure to recognize targets presented in the center rather than in the periphery (Vallejo et al., 2016), longer or shorter gaze duration compared with controls when reading high, medium, and low predictable sentences (Fernandez et al., 2014a,2015a,2015b; Galetta et al., 2017), more fixations, and altered visual exploration while reading sentences, some with lack of context predictability (Fernandez et al., 2013, 2014b,2015b). However, the impaired performance of patients may reflect subject responses to cognitive impairment hampering task performance that has already appeared but not as a manifestation of cognitive decline to eye movements *per se*; the very fact that patients cannot perform the required task properly is reflected in the way patients look at the targets.

While such tasks require the active cooperation of the subjects and prior knowledge from the context, eye-tracking can also provide a sensitive measure of cognitive impairment by directly looking at the exploration patterns of the eye movements themselves without explicitly giving instructions. For example, by observing the pattern of looking at presented images, we can visualize the abnormalities in attentional deployment (Delazer et al., 2018). Evidence is accumulating that the eye movement pattern can be an effective biomarker of AD. Daffner et al. (1992) compared eye movements of AD patients and control subjects while looking at pairs of regular (“congruous”) and irregular (“incongruous”) figures. They found that control subjects spend more time viewing irregular figures than regular ones, while AD patients viewed regular and irregular figures equally. This result suggested that AD patients’ gaze reflects diminished curiosity, which can be evaluated by their eye movements. Mosimann et al. (2004) found that, when AD patients are reading clocks, their visual explorations were less focused, with fewer fixations inside the informative regions of interest (ROIs), compared to control subjects. Oyama et al. (2019) developed a cognitive assessment utilizing eye-tracking technology that showed good diagnostic performance in detecting patients with dementia, mild cognitive impairment (MCI), and control subjects. Their results demonstrate that observation of the eye movement patterns in visual exploration can be used as a biomarker of AD, although the eye movement patterns may just be the result of cognitive impairment, with subjects responding with their eyes instead of verbally. Thus, any abnormal responses in those tasks may be equal to giving wrong verbal answers and/or responses to having made an inappropriate response or failure to respond to the question.

For the early diagnosis of cognitive impairment, subtle abnormal exploration patterns should be detected at the very beginning of the cognitive impairment, even before overt deterioration becomes apparent. In addition to memory disorder, patients with AD are known to exhibit various cognitive disorders, including a decline in visuospatial cognition, which is reflected in eye movement patterns. Even when task performance requiring a verbal response is still within the normal range, subtle cognitive impairment can manifest in the form of abnormal attentional deployment, slowed visual information processing speed, and the increased cognitive effort of the subjects when performing the tasks. Based on these premises, we have developed new methods to test visuospatial cognition by eye-tracking (Matsumoto et al., 2011a,b, 2012; Matsuda et al., 2014; Tokushige et al., 2018): a visual memory task and a visual search task. In the visual memory task, the eye movements are recorded while subjects tried to memorize figures in order to visualize the fixation pattern relating to which part of the figure was frequently seen by the subject. In the visual search task, the eye movements while searching for a target among an array of distractions is recorded, which indicates how efficiently the subject can search for the target. Contrary to clinically used cognitive tests requiring verbal responses, which subjects with dementia often fail to perform properly, these are tasks that these subjects can perform to some extent with relative ease. Eye tracking successfully revealed the abnormal visual processing in these subjects, which was correlated with the degree of cognitive decline. This method is expected to be useful in the early detection of cognitive decline in at-risk subjects in the early stage of AD by revealing multiple aspects of cognitive impairment, such as attentional allocation, visual processing, and task engagement.

It is important to note that eye tracking parameters in the visual memory and search tasks cannot be used as a direct measure of impaired cognitive (visuospatial) processing alone, or should be considered reflections of visuospatial processing, visual attention and memory. In this study, to enable early detection of cognitive decline, we aimed to evaluate the earliest changes in eye tracking patterns that occurred at the earliest stage of cognitive decline, rather than differentiating the components one by one, and studied whether these occur in correlation with the cognitive decline.

## Materials and methods

### Participants

Sixteen AD patients and sixteen control subjects were recruited (Table 1). Between these groups, there was no significant difference in the age (T-test, *p* = 0.914) or in the gender (Fisher’s test, *p* = 0.722). The MMSE score was significantly lower in the AD group (T-test, *p* < 0.001). The diagnosis of AD was clinically made as probable AD by brain MRI and single photon emission computed tomography (SPECT), based on the revised NINCDS (National Institute of Neurological and Communicative Disorders and Stroke)-ADRDA (Alzheimer’s Disease and Related Disorders Association) criteria (Dubois et al., 2007). Since positron emission tomography (PET) could not be performed, it was substituted by the results of SPECT. Patients using bilateral intraocular lenses were excluded because light reflection on the lens surface disturbed eye tracking. Patients who could not perform the tasks or could not follow the instructions to perform the tasks due to advanced cognitive decline or loss of visual acuity were also excluded. Written informed consent to participate in the study was obtained from all participants before the experiments. The experimental procedures were approved by the Ethics Committee at the University of Tokyo (No. 2411), which was conducted in accordance with the ethical standards of the Declaration of Helsinki.

**TABLE 1:** Summary of subjects in the visual memory task.

	AD (*n* = 16)	Control (*n* = 16)	*p*-value
Age	79.1 ± 7.9	79.4 ± 4.6	0.914
Male/female	8/8	10/6	0.722
MMSE	17.7 ± 5.3	26.9 ± 2.4	<0.001

### Visual memory task

**FIGURE 1:**
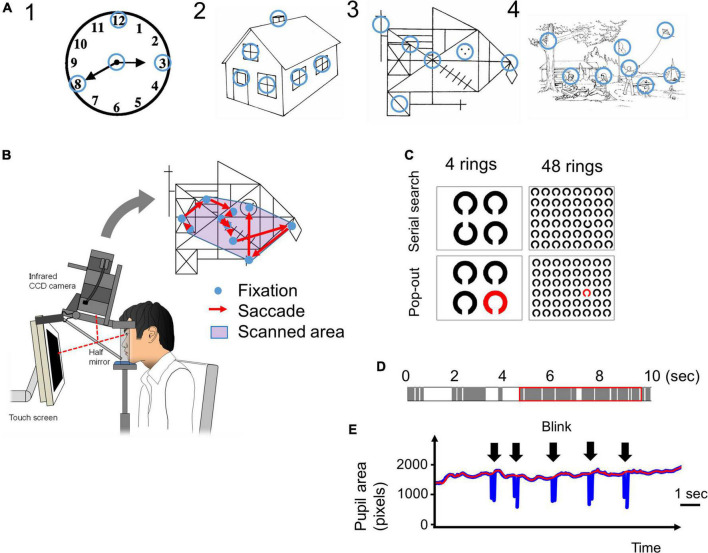
**(A)** The four images presented in the visual memory task. Blue circles, which are not visible to the subjects, indicate the informative regions of interest (ROIs) that characterize the image. We determined the locations of these informative regions based on previous studies (Mosimann et al., 2004; Matsumoto et al., 2011b). The radius of these ROIs was 1.9 deg in visual angle. **(B)** The eye-tracking system. A 17-inch computer display is placed 50 cm in front of the subject’s eyes. The images are presented on the display, and the subject’s eye movements (fixations and saccades) while looking at the image are recorded by the video-oculography camera (Eyelink 1000^®^). The scanned area is defined by the convex envelope of all fixation points. **(C)** The images used in the visual search tasks. In the serial search task, the target Landolt ring is placed upwards and others are placed downwards. In the pop-out task, the target ring is red and the others are black. In both tasks, the number of rings was 4 or 48. **(D)** An example of time distribution in 10 s while a subject is looking at an image. The gray bars indicate the fixation periods, and the white bars indicate other times (saccades, blinks, face displacements, and so on) that indicate failure of fixation. The red rectangle indicates the 5 s that include the longest total time duration of fixations. **(E)** An example of a pupil size plot during the visual search task. Pupil size data (blue line) cannot be recorded during blinks (arrow). The red line is the upper envelope of pupil size data, which is not influenced by saccades.

The procedure has been previously described by Matsumoto et al. (2011a,b, 2012). Briefly, subjects viewed images of various complexities [Figure 1A, Image 1: a clock in Western Aphasia Battery (WAB), Image 2: a house in WAB, Image 3: Rey-Osterrieth Complex Figure (Osterrieth, 1944), Image 4: a landscape in WAB] on a 17-inch computer display (1024 × 768 pixels) placed 50 cm in front of their eyes for 10 s trying to memorize them, and after the images disappeared from the screen, drew the images on a paper based on their memory. Eye movements made during task performance were recorded with video-oculography Eyelink1000^®^ (SR Research, Mississauga, Ontario, Canada) (Figure 1B).

The ROIs were defined as visually salient positions that have important information in the figure and on which control subjects most frequently fixated according to the methods of Matsumoto et al. (2011a,2012); they are indicated by blue circles in Figure 1A (the blue circles were invisible to the subjects). The radius of the ROI, 50 pixels in the display, corresponded to 1.9 deg in visual angle.

The accuracy of the recalled pictures drawn by the subjects was scored with the following drawing scores: images 1 and 2 (both 0–5 points) were assessed by scores in WAB, and image 3 (0–36 points) was assessed based on Osterrieth (1944). There was no scoring system for image 4, and the number of items the subjects drew was his/her drawing score. For example, if someone drew only a man and a dog, the drawing score was 2.

### Visual search tasks

We employed two visual search tasks, serial search and pop-out, as described by Matsuda et al. (2014), based on the methods of Treisman (1998). Briefly, from among an array of multiple Landolt rings arranged on the display, subjects searched for a sole target ring (target ring) with a different orientation (serial search) or different color (pop-out search) from the other rings. In the serial search task, the cleft of the target Landolt ring was oriented upward, whereas the other non-target Landolt rings were oriented downward. In the pop-out search task, the target Landolt ring was red while the non-target Landolt rings were black (Figure 1C). After detection of the target ring, the subjects were instructed to gaze at it and to press the button immediately, at which point the image disappeared from the screen. The time limit for button press was 60 s after the start of image presentation. The trial was automatically terminated in 60 s and the task proceeded to the next trial if no button was pressed.

The number of Landolt rings on the display was either 4 or 48. The number of Landolt rings determines the difficulty of the serial search task. When there are only 4 rings, it is easy to find out the target (there is even no need to search for it), but when there are 48 rings, the search is difficult so that subjects have to look at the rings one by one. Thus the effect of cognitive decline was expected to appear in the 48 ring task, but not in the 4 ring task. The selection of the numbers of stimuli were based on previous reports (Treisman and Gelade, 1980; Matsuda et al., 2014). The reason for the large difference in number of the stimuli, 4 and 48, was because we wanted to detect the slightest cognitive decline through the difference in task performance; a moderate difference in number of stimuli may result in a failure to detect the difference between groups (cognitive impaired and control subjects). By comparing the results of 4 rings task and 48 rings task, we would be able to detect the effect of dementia clearly.

Each trial was repeated 10 times with the target location randomly assigned every time. Eye movements made during task performance were recorded with video-oculography using Eyelink1000. Among the subjects participating in the visual memory task, the data of four AD patients were excluded, since they could not complete the visual search task properly, and thus the data from twelve AD patients and sixteen control subjects (Table 2) were analyzed. Between these groups, there was no significant difference in the age (T-test, *p* = 0.785) and in the gender (Fisher’s test, *p* = 0.702). The MMSE score was significantly lower in the AD group (T-test, *p* < 0.001).

**TABLE 2:** Summary of subjects in the visual search task.

	AD (*n* = 12)	Control (*n* = 16)	*p*-value
Age	78.7 ± 7.9	79.4 ± 4.6	0.785
Male/female	6/6	10/6	0.702
MMSE	19.3 ± 3.3	26.9 ± 2.4	<0.001

### Data processing

We used the Eyelink Data viewer software (ver1.3.37, SR Research, Mississauga, Ontario, Canada) for data processing. During the visual memory task, images were presented for 10 s for the subjects to memorize, but the pupil data could not necessarily be obtained for the entire 10 s. This was because eye tracking was sometimes truncated as a result of a variety of events, and there were periods during which the gaze and pupil size data were lost within the 10 s. These truncating events included blinks that occluded visual inputs from the camera, and displacement of the face sometimes interrupted the eye tracking transiently. Furthermore, the gaze of some subjects, especially AD patients, occasionally went outside of the display during the trials, possibly due to distraction. Figure 1D shows an example of the composition of a 10 s period during which an image was presented. The gray bars indicate periods of fixation, and the white bars indicate time periods other than fixation with pupil data loss (these included saccades, blinks, face displacements, and so on). For analysis, we took the continuous 5 s period that contained the longest summed time duration of fixation periods among the entire presentation period of 10 s (red rectangle in Figure 1D). During this 5 s period, the density of the fixation period is higher than that during the entire 10 s period, and it excludes the time period with the least pupil data. However, despite selecting the period with the longest summed fixation period, some subjects could not fixate on the images for an adequately long duration. The data of subjects who could not fixate on the screen for 3 s within the selected 5 s (in other words, when the summed durations of gray bars in the red rectangle in Figure 1D were less than 3 s) were excluded.

The total number and mean amplitude of saccades, duration of fixation, proportions of the scanned area, dwell time within ROIs, and number of ROIs fixated were measured for each figure. The subjects were considered to have fixated a ROI if the gaze (i.e., fixation point on the screen) entered the ROI circle (Figure 1A) with the radius of 50 pixels (1.9 deg in visual angle). The proportion of the scanned area was calculated by dividing the area of a minimal convex polygon enclosing all of the fixation points (Figure 1B) by the entire area of the screen. The dwell time within ROIs represents the summed duration of fixations made within the ROI during image presentation. The number of focused ROIs was the number of ROIs (Figure 1A) that the subjects’ gaze entered at least once during image presentation. These parameters were calculated individually for every session in all subjects.

For the visual search tasks, in addition to the saccade number and amplitude, we measured the frequency of saccades (number of saccades made per second, counts/s) and the search time (ms), which was the time required for the subjects’ eyes to finally reach the target. We judged the eyes to have reached the target when the fixation point on the display approached the center of the target, within a radius twice as large as that of the target ring. Some subjects fixated the target more than once (target re-fixation); the gaze of subjects entered the circle twice as large as the target ring, left the circle, and re-entered the circle again, which occurred more than once during the search. In such cases, the last time the fixation point entered the circle was regarded as the search time.

### Pupil size analysis

From the eye tracking data in the visual search task, we extracted the time-varying data of the pupil size at a sampling rate of 1,000 Hz. The pupil size was given by the count of pixels included within the pupil ellipse detected by the eye tracking camera. An example of a pupil size plot is shown in Figure 1E (blue line). Whenever a blink occurs, the pupil is hidden and the pupil size captured by the camera will be lost; as such, the curve temporarily goes to zero, producing a blink artifact in the pupil size plot (arrow). To compensate for this artifact by interpolating for the lost data, we made upper envelopes of the pupil size plot (Figure 1E, red line). Then we averaged the pupil size for the duration of each figure presentation in each subject. Pupil size in each trial was expressed as a ratio to the average pupil size in the first trial in each subject (pupil size ratio). These analyses of pupils were performed only for the data of serial search task, but not for the visual memory or pop-out tasks.

### Statistical assessment

The parameters of visual search were compared between AD and control subjects. In the visual memory task, for each parameter (number of saccades, saccade amplitude, duration of fixation, scanned area, dwell time in ROI, and number of focused ROIs), repeated measures two-way analysis of variance (ANOVA) was performed with the group (AD vs. control) as a between-subject factor and image (images 1–4) as a within-subject factor. Where necessary, the Greenhouse-Geisser correction was used to evaluate non-sphericity. The correlation between these parameters and the MMSE score or drawing score was assessed by the Pearson correlation coefficient with Bonferroni correction. If there was a significant difference between AD and control groups in a saccade parameter, we performed receiver operating characteristic (ROC) analyses to determine whether these parameters could efficiently differentiate these groups at certain cut-off values.

In the visual search task, each parameter (search time, number of saccades, saccade frequency, and saccade amplitude) was averaged across the images (1–10) for each subject, and compared between AD and control subjects using the *t*-test. Again, the correlation between these parameters and the MMSE score was assessed by the Pearson correlation coefficient with Bonferroni correction. As in the visual memory task, if there was a significant difference between AD and control groups in a saccade parameter, we also performed ROC analyses to determine whether these parameters could effectively differentiate these groups.

The pupil size ratio in the visual search task (serial search), averaged for images 1 to 10, was compared between AD and control subjects using the *t*-test. The correlation between the pupil size ratio and the MMSE score was assessed by the Pearson correlation coefficient. As in the experiments above, if there was a significant difference between these groups, we performed ROC analyses to determine how efficient this parameter was for the differentiation of these groups.

## Results

### Visual memory task

**FIGURE 2:**
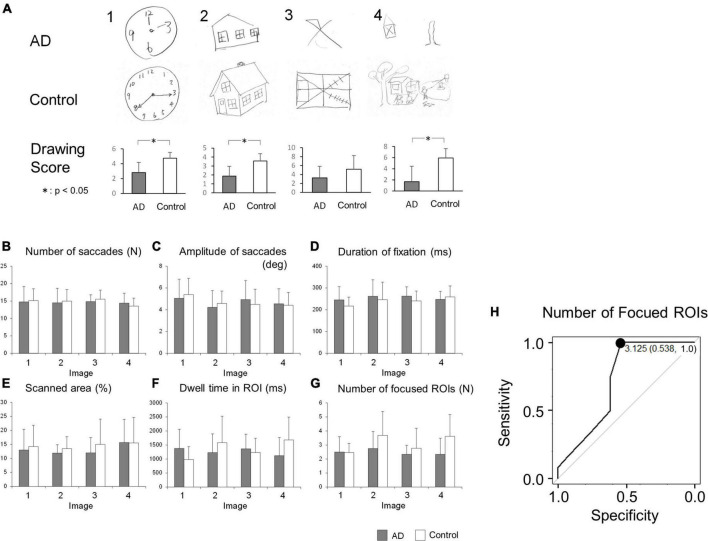
**(A)** The result of image drawing of the visual memory task. Examples of images drawn by AD patients and control subjects are shown. The drawing scores tended to be lower in AD patients. **(B–G)** Comparison of saccade parameters in the visual memory task between AD patients and control subjects. By repeated measures two-way ANOVA, the difference between these groups was significant only in panel **(G)** the number of focused ROIs (see the text). **(H)** The ROC analysis of the number of focused ROIs, averaged for images 1-4. When the cut-off was set to 3.125, the sensitivity was 1.0 and the specificity was 0.538. *p < 0.05.

The results of the visual memory task are shown in Figure 2. Figure 2A shows a representative example of reproduced figures. AD patients tended to be poorer at reproducing any of the images than control subjects. Mann-Whitney’s U-test showed that the drawing scores were significantly lower in AD patients for the images (bottom figures of Figure 1; Image 1, AD 2.81 ± 1.38, control 4.75 ± 0.77, *p* < 0.001; Image 2, AD 1.88 ± 1.09, control 3.56 ± 0.81, *p* < 0.001; Image 4, AD 1.69 ± 1.66, control 5.94 ± 2.77, *p* < 0.001) except for image 3, in which the difference showed a trend (AD 3.25 ± 2.58, control 5.19 ± 3.04, *p* = 0.057). For all of the presented images, the drawing score showed a significant positive correlation with MMSE score (Image 1: *R* = 0.621, *p* < 0.001, Image 2: *R* = 0.602, *p* < 0.001, Image 3: *R* = 0.548, *p* = 0.00116, Image 4: *R* = 0.682, *p* < 0.001).

The number of saccades while viewing the images is compared in Figure 2B. Repeated measures two-way ANOVA indicated that the effect of the group (*F*[1, 23] = 0.0482, *p* = 0.828), the image (*F*[3, 69] = 0.865, *p* = 0.464), or their interaction (*F*[3, 69] = 0.407, *p* = 0.749) was not significant, showing that both AD patients and control subjects made a similar number of eye movements (saccades) while viewing the images.

Figure 2C compares the saccade amplitude while viewing the images. The effect of the image (repeated measures two-way ANOVA: *F*[3, 69] = 3.11, *p* = 0.0320) was significant, while the effects of the group (*F*[1, 23] = 0.0054, *p* = 0.942) and their interaction (*F*[3, 69] = 0.884, *p* = 0.454) were not, which suggested that the saccade amplitude depends on the image presented; this did not differ between AD patients and control subjects.

Figure 2D compares the average duration of fixation between the AD patients and control subjects. The effect of the group (repeated measures two-way ANOVA: *F*[1, 23] = 0.640, *p* = 0.432), the image (*F*[3, 69] = 1.60, *p* = 0.197), or their interaction (*F*[3, 69] = 1.02, *p* = 0.390) did not reach significance. This implied that the duration of fixation in AD patients was not significantly different from that of control subjects, regardless of the image viewed.

Figure 2E depicts the total area scanned by the gaze while subjects viewed the images. The scanned area of AD patients appeared to be smaller than that of control subjects. However, by repeated measures two-way ANOVA, the effect of the group (repeated measures two-way ANOVA: *F*[1, 23] = 0.760, *p* = 0.393), the image (*F*[3, 69] = 0.839, ε = 0.681, *p* = 0.441), and their interaction (*F*[3, 69] = 0.231, ε = 0.681, *p* = 0.799) did not reach significance, showing that the total area scanned by the subjects’ gaze of AD patients was comparable to that of control subjects across the images presented.

We also compared the dwell time of the gaze within the ROIs (Figure 2F), which refers to the length of time the subjects’ gaze stayed within the informative ROIs of each image (blue circles in Figure 1A). Repeated measures two-way ANOVA showed that the effects of the group (*F*[1, 23] = 0.382, *p* = 0.543) or image (*F*[3, 69] = 0.704, *p* = 0.553) were not significant, while their interaction (*F*[3, 69] = 2.99, *p* = 0.0370) was. This suggested that AD patients and control subjects differed in their dwell time within ROIs of different images; AD patients’ gaze spent more time within the ROIs while viewing simple images while control subjects’ gaze dwelled more in the ROIs while viewing complex images, as depicted in the bar graph for images 1 and 4 in Figure 2F.

The number of ROIs fixated, that is, the number of informative ROIs that their eyes entered within a certain distance (see section “Materials and methods”) at least once, is compared in Figure 2G. Here, the effect of the group (*F*[1, 23] = 5.42, *p* = 0.0291) reached significance, but the effect of the image (*F*[3, 69] = 2.21, ε = 0.717, *p* = 0.117) or their interaction (*F*[3, 69] = 1.50, ε = 0.717, *p* = 0.234) did not. This indicated that AD patients look at a smaller number of informative ROIs than control subjects do. AD patients collect information from a smaller number of ROIs within the images compared to control subjects, although subjects in both groups explored each image for the same duration of time and their eye fixation time and saccade frequency made per unit time was similar (Figures 2B, D).

Since AD patients tended to focus on a smaller number of informative ROIs than control subjects, we tested whether this feature could be used to differentiate AD patients from control subjects by ROC analysis (Figure 2H). When the cut-off value was set to 3.125, the sensitivity was 1.0 and the specificity was 0.538.

Table 3 shows the correlation between the drawing score and the saccade parameters. We performed Bonferroni correction with the significance level of *p* value set at 0.003125, i.e., 0.05 divided by the number of comparisons 16. According to this criterion, none of the parameter significantly correlated with the drawing score. Table 4 shows the correlation between the MMSE score and saccade parameters; again, there were no significant correlations after Bonferroni correction (*p* > 0.003125).

**TABLE 3:** The correlation between the drawing score and saccade parameters in the visual memory task.

	Image 1		Image 2		Image 3		Image 4	
	*R*	*p* value	*R*	*p* value	*R*	*p* value	*R*	*p* value
Number of saccades	0.0685	0.714	-0.026	0.893	0.0935	0.623	-0.162	0.400
Amplitude of saccades	–0.0739	0.693	0.00641	0.974	0.00244	0.99	0.0623	0.748
Duration of fixation	–0.269	0.143	-0.0773	0.690	-0.158	0.403	0.240	0.210
Scanned area	0.137	0.462	0.462	0.0116	0.204	0.278	0.0707	0.716
Dwell time in ROI	–0.342	0.0598	0.242	0.205	0.0529	0.781	0.269	0.158
Number of focused ROIs	–0.0985	0.598	0.334	0.0769	0.254	0.176	0.369	0.0486

**TABLE 4:** The correlation between the MMSE score and saccade parameters in the visual memory task.

	Image 1		Image 2		Image 3		Image 4	
	*R*	*p* value	*R*	*p* value	*R*	*p* value	*R*	*p* value
Number of saccades	–0.0346	0.854	-0.0965	0.619	0.00824	0.966	-0.334	0.0761
Amplitude of saccades	0.0784	0.675	0.0774	0.690	0.0262	0.891	0.0421	0.828
Duration of fixation	0.0471	0.801	0.0456	0.814	-0.0638	0.737	0.344	0.068
Scanned area	–0.220	0.234	0.158	0.414	0.029	0.879	0.0353	0.856
Dwell time in ROI	–0.298	0.104	0.231	0.228	-0.165	0.384	0.233	0.225
Number of focused ROIs	–0.306	0.0936	0.348	0.0641	0.111	0.558	0.315	0.096

### Visual search task

**FIGURE 3:**
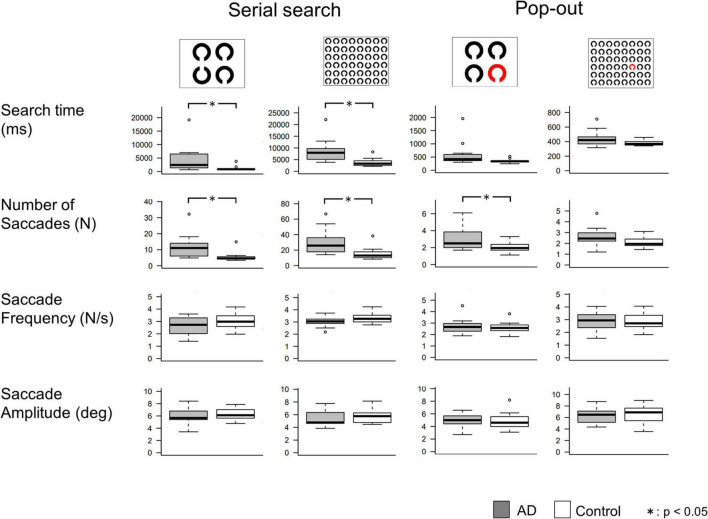
Comparisons of saccade parameters in the visual search tasks. *T*-test shows that AD patients required significantly longer search time and more saccades than control subjects, mainly in the serial search task. *p < 0.05.

The results of the visual search task are shown in Figure 3, and the results of the *t*-test are summarized in Table 5. In the serial search task with 4 rings, AD patients required significantly more time and a larger number of saccades to search for the target, while the saccade frequency and amplitude showed no significant difference between AD patients and control subjects. A similar trend was also noted in the serial search task with 48 rings, where AD patients required significantly more time and number of saccades although there was no significant difference in the saccade frequency or amplitude compared to control subjects.

**TABLE 5:** The *p* values of T-test of saccade parameters between AD patients and control subjects in the visual search task.

	Serial search		Pop-out	
	4 rings	48 rings	4 rings	48 rings
Search time	0.0383*	0.0059*	0.0839	0.0823
Number of saccades	0.016*	0.00878*	0.0255*	0.0780
Saccade frequency	0.169	0.0773	0.563	0.801
Saccade amplitude	0.515	0.389	0.943	0.571

*p < 0.05.

In the pop-out task with 48 rings, there was no significant difference in the search time, saccade counts, frequency, or amplitude between AD patients and control subjects. In the pop-out task with 4 rings, AD patients required more saccades to arrive at the target, but there was no significant difference in the search time, saccade frequency, or amplitude between the two groups.

Table 6 shows the correlations between the MMSE score and saccade parameters in the visual search task. Here again, we performed Bonferroni correction with the significance level of *p* value set at 0.003125. The search time and the number of saccades showed a significant negative correlation with the MMSE score in the serial search tasks (*p* < 0.001), but not in the pop-out tasks. Saccade frequency and amplitude did not correlate significantly with the MMSE score in any of the tasks.

**TABLE 6:** The correlation between the MMSE score and the saccade parameters in the visual search task.

	Serial search				Pop-out			
	4 rings		48 rings		4 rings		48 rings	
	*R*	*p* value	*R*	*p* value	*R*	*p* value	*R*	*p* value
Search time	−0.664	<0.001*	−0.752	<0.001*	−0.298	0.123	−0.371	0.0521
Number of saccades	−0.671	<0.001*	−0.738	<0.001*	−0.348	0.0695	−0.194	0.323
Saccade frequency	0.289	0.136	0.134	0.496	0.0342	0.863	−0.0427	0.829
Saccade amplitude	0.067	0.735	0.242	0.215	−0.220	0.260	−0.0397	0.841

*p < 0.003125 (Bonferroni correction).

**FIGURE 4:**
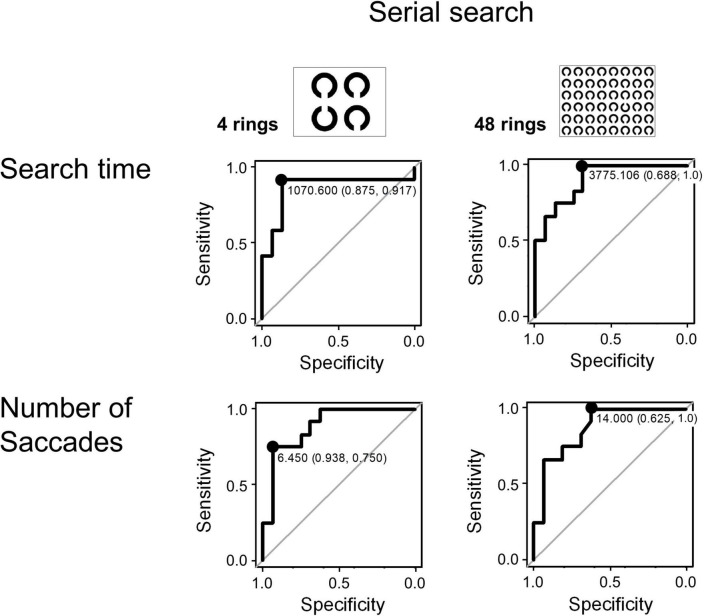
The ROC analysis of search time and number of saccades in the visual search task (serial search).

Since the search time and the number of saccades in the serial search task showed significant differences between the AD and control groups, we performed ROC analyses to determine whether these parameters can effectively differentiate the groups by setting certain cut-off values. Figure 4 shows the results of the ROC analyses. Whereas both the search time and the number of saccades effectively differentiate these groups, the search time performed better. In the 4 ring task, when the cut-off time was set at 1070.6 ms, the sensitivity was 0.917 and the specificity was 0.875. In the 48 ring task, when the cut-off time was set at 3775.106 ms, the sensitivity of the search time was 1.0 and the specificity was 0.688. The results of ROC analysis on the number of saccades were as follows: in the 4 ring task, the sensitivity was 0.75 and the specificity was 0.938 when the cut-off value was 6.45; and in the 48 ring task, the sensitivity was 1.0 and the specificity was 0.625 when the cut-off value was 14.

### Pupil size analysis

**FIGURE 5:**
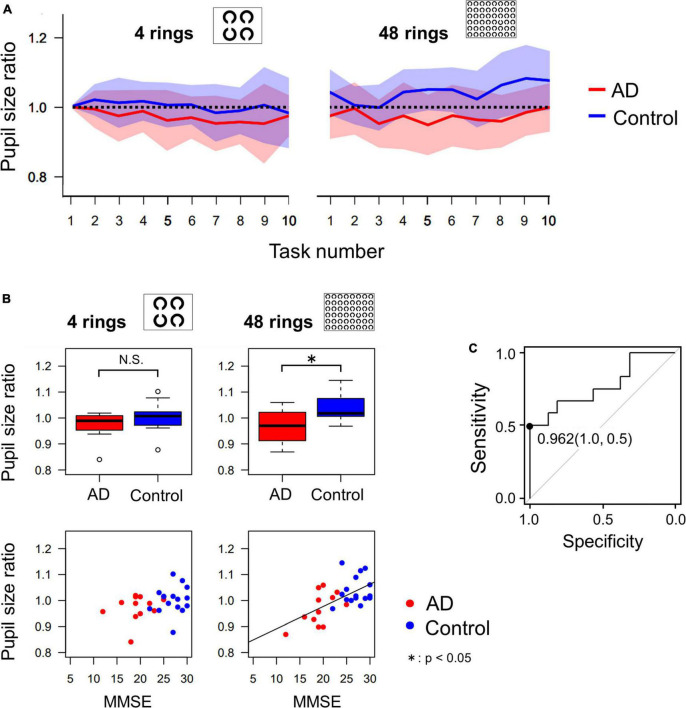
**(A)** The change of pupil size ratio during the serial search task as the task progressed. The red and blue lines indicate the average data of AD patients and control subjects, respectively, and the width of the color bars around the red or blue lines indicate their standard deviations. The pupil dilatation of AD patients tended to be less than that of control subjects, especially in the 48 ring task. **(B)** Comparison of pupil size ratio averaged across all 10 images (4 rings or 48 rings) between AD and control groups, which shows that pupil dilatation of AD patients is significantly less than that of control subjects in the 48 ring task, by *t*-test. The correlation between the MMSE score and the pupil size ratio was significant only in the 48 ring task. **(C)** The ROC analysis of the pupil size ratio in the 48 ring task. When the cut-off value is set to 0.962, the sensitivity was 0.5 and the specificity was 1.0. *p < 0.05.

Figure 5A shows the change in pupil size in AD patients and control subjects in sequential tasks, which was expressed as a ratio (pupil size ratio) calculated by dividing the mean pupil size during the current by its baseline value, that is, the mean pupil size in the first task for each subject. The pupil size ratio of AD patients was lower than that of control subjects, especially when the number of Landolt rings was 48.

Figure 5B compares the mean pupil size ratio of AD patients and control subjects in the 4 and 48 ring tasks (upper figures) and shows the correlation between the pupil size ratio and the MMSE score (lower figures). In the 4 ring task, the pupil size ratio did not differ significantly between AD patients and control subjects (*t*-test, *p* = 0.135). There was no significant correlation between the pupil size ratio and the MMSE score (*R* = 0.338, *p* = 0.0788). However, in the 48 ring task, the pupil size ratio of AD patients was significantly smaller than that of control subjects (*t*-test, *p* = 0.00569). There was a significant positive correlation between the pupil size ratio and the MMSE score (*R* = 0.608, *p* < 0.001).

Since the pupil size ratio in the 48 ring serial search task differed significantly between the AD and the control group, we performed ROC analysis to differentiate these groups using this parameter (Figure 5C). When the cut-off value was set at 0.962, the sensitivity was 0.5, the specificity was 1.0, and the area under the curve was 0.771.

## Discussion

In this study, we analyzed the eye movements of AD patients and control subjects performing visual memory and search tasks. In the visual memory task, while saccade parameters such as the frequency or amplitude of saccades did not differ significantly between the two groups, AD patients fixated fewer informative ROIs in the images, even when there was sufficient time to do so. This resulted in a poorer recall of the memorized images, which deteriorated with cognitive decline. In the visual search task, AD patients needed to make more saccades and spent more time before finding the target than control subjects, whereas, again, the saccade frequency (per unit time) and amplitude did not differ significantly from control subjects. The pupil size analysis in the visual search task showed that pupils of control subjects increased in size accumulatively as they performed consecutive trials of the visual search task with 48 rings, although this was not observed in AD patients. Taking these measures together, our visual scanning task could successfully characterize and detect the dysfunction of visuospatial processing in AD patients with high sensitivity and specificity, and could thus become a new biomarker for the early diagnosis and evaluation of cognitive decline.

### Impaired deployment of attention and reduced recall of memory in AD patients

We gather visual information from the outer world by making repeated saccades and fixations on a scene, paying particular attention to the informative areas. Thus, deploying visual attention efficiently over a scene, whether bottom-up or top-down, is important to collect visual information from the scene. In the visual memory task, AD patients became increasingly impaired at recalling the line drawings as cognitive decline progressed; AD patients also tended to view a smaller number of informative, visually salient ROIs in the images during the visual memory task compared to control subjects (Figure 2G).

Perceptual salience may emerge mainly under low task constraints, such as during free viewing or scene memorization, as used in this task. For example, when we look at someone’s face, our fixation points are concentrated on the eyes, nose, or mouth, and the contour of these components, but not much on the skin in between (Yarbus, 1967). In exploring a scene, subjects may selectively direct attention to objects using both bottom-up, image-based saliency cues or top-down, task-dependent cues (Itti and Koch, 2001). In the bottom-up mode, the gaze is drawn automatically to visually salient regions of the scene. The visually salient and less salient positions, respectively, form a peak and trough in the salience map, and the visually salient locations draw bottom-up attention and saccades toward that location during visual exploration. Instead, in the top-down mode, the ROIs are not visually salient, and the viewer would have to actively deploy their gaze, and possibly their attention as well, over the scene. If subjects fail to pay attention to the informative areas, they would fail to collect visual information from the outer world efficiently, and hence have a lower drawing score. The duration of fixations on individual objects within a scene is shown to affect later recall of the same object (Chapman, 2005).

The inability of AD patients to pay attention to the informative areas of what they are viewing has been reported using a clock reading task. Mosimann et al. (2004) demonstrated that the visual exploration of AD patients was less focused, with fewer fixations on the task-relevant ROIs (the numbers on the clock surface pointing to the time of the clock) compared to control subjects. Normally, while visual search does not leave a memory of its trajectory, an inhibition-of-return mechanism acts on the already-selected locations, and should promote looking at unseen locations of the images (Bridgeman et al., 1975; Horowitz and Wolfe, 1998). Their study and ours both demonstrated that AD patients are poorer at allocating attention broadly to all of the informative areas, even when they had sufficient time to do so, suggesting a restriction in attentional deployment.

AD patients were taking AD medicine (mainly acetylcholinesterase inhibitors such as donepezil). Although the effect of AD drugs on visual memory or search tasks remains to be clarified, a previous study showed that donepezil improves visual working memory performance (Reches et al., 2014). In view of this result, the medicines may have improved the performance of visual memory or search tasks. Even considering this potential drug effect, the performance of the patients was demonstrated to be lower than the age-matched control subjects.

The performance of AD patients may be compared to Parkinson’s disease (PD) patients who also showed reduced area of scanning when viewing similar scenes for later recall. PD patients were more restricted in the area scanned within the image, which resulted from reduced saccade amplitude and lower frequency of saccades compared to control subjects (Matsumoto et al., 2011b; Terao et al., 2013). Notably, the performance of PD patients characterized by reduced saccade frequency and amplitude, presumably caused by basal ganglia dysfunction, was most deviated from control subjects for less complex figures, and approached control performance for more complex figures. This improvement in saccade performance was explained by “ocular” kinesie paradoxale, in which the reduced scanning area became closer to control since PD patients could use the visual cues contained in the figures as a guide to improve the frequency and size of ocular movements (Matsumoto et al., 2011b,^2012^), and the smaller scanning area of the images became comparable to control subjects. In contrast, the saccade amplitude and frequency in AD patients was similar to that of control subjects, suggesting that the underlying pathophysiology for the reduced scanning area was different from that of PD patients. Thus, the restriction in attentional deployment may result from dysfunction of the visuospatial processing in the cerebral cortex and working memory in patients, rather than from abnormal basic saccade control by the basal ganglia and/or brainstem. The saccade amplitude of AD patients was not significantly different from that of control subjects (Figure 2C).

ROC analysis for the visual memory task showed that the number of ROIs viewed showed a high sensitivity but low specificity for cognitive decline (Figure 2H), which can be used as a screening method for AD diagnosis, but not for ruling out subjects with a preserved cognitive functions.

### Inefficient visual processing in AD patients

In the serial but not pop-out visual scanning task, AD patients required more saccades and more time than control subjects before they could detect the target, in both the 4 and 48 ring tasks (Figure 3). In AD patients, the search time and number of saccades correlated negatively with the MMSE score, that is, it increased with the progression of cognitive decline (Table 6), whereas saccade frequency and amplitude stayed almost constant with disease progression. Thus, the increased search time with increased number of saccades indicated that visual search was inefficient in AD patients. These results are consistent with those of previous studies that showed a relatively preserved single-feature search as opposed to a more impaired feature-conjunction search in AD (Cormack et al., 2004; Hao et al., 2005; Viskontas et al., 2011; Landy et al., 2015; see Ramzaoui et al., 2018 for review). These findings translate here to preserved bottom-up, involuntary engagement of attention (pop-out task) and deficits in top-down, voluntary attention (serial search task).

As in the visual memory task, subjects repeatedly made saccades and fixations during the visual search task to search for the target, but in the visual search task, guidance was also made largely from bottom-up perceptual guidance. The visual search strategy was initially likened to a serial scanning process in which task subjects must judge what he/she is currently looking at every time they look (overt attention) or the covert attentional window which captures the object included in the search array, either the target or the distractor (Findlay and Gilchrist, 2005). If the subjects recognize that the object that they overtly captured by eye movement or covertly scanned by the attentional window is the target, the search is terminated without further saccade processing. On the other hand, if the subjects judge that they are looking at the distractor, they have to start programming another saccade to the next potential target. In this successive process, the visual search may occur in three stages: (1) judgment of the object as the target or the distractor, (2) seeking out the candidate location for the next potential saccade, and (3) programming and initiation of the saccades. This model posits a covert attentional window (if not the gaze or central visual field themselves) that is scanning the array of objects, including the target and the distractors. However, this model proved to be unable to explain all of the visual search findings to date (see Findlay and Gilchrist, 2005 for review). First, there does not appear to be any apparent discontinuity in the search time between serial and pop-out searches. Second, for large search arrays, there was a close correlation between search time and the number of saccades made before the target is located, and it appears implausible for a rapid attentional scan to be operative with each fixation to seek out the candidate location for the next saccades’ destination within the fixation period of around 200 ms. Thus, the prevailing view is a hybrid of parallel processing within each fixation with serial saccade scanning. Items closer to fixation and similar to the target will tend to have higher salience in the saliency map and be more likely to be selected as a next target. If the prolonged search time of AD patients comes from impaired saccade programming, the saccade frequency of AD patients must differ from that of control subjects, which was not the case (Figure 3). The longer search time of AD patients may thus arise from the defect in constructing a proper salience map rather than the serial overt or covert attentional scan being defective, because there was no significant difference in the saccade parameters (frequency and amplitude) between AD patients and control subjects.

Problems in low-level visual processes have been reported among the earliest symptoms in AD (Armstrong, 2009; Kirby et al., 2010; Verheij et al., 2012). While AD patients show high reliance on guidance from perceptual features (or saliency, i.e., preserved performance in the pop-out task), they exhibit difficulty in locating targets when target contrast or texture difference with the background was reduced, as in the case of a serial search task with many objects. However, understandably, these tasks tax the cognitive function of AD patients and the difficulty of accomplishing the task itself may affect the gaze pattern during visual search. Our present task did not require much effort on the part of patients, and most of the AD patients could complete the task without much difficulty. Thus, behavioral change was not the result of the subjects being unable to search for the target, but rather, was due to delayed visual scanning caused by inefficient visual processing in the patients.

The inefficient visual search was already compromised in AD patients at an early stage. Remarkably, ROC analysis showed that search time and number of saccades made until target detection in the 48 ring task achieved a high sensitivity (100%, Figure 4) and specificity. These measures also showed a significant correlation with the MMSE score (Table 6). Because of its high sensitivity and specificity, the search time and saccade number in the visual search task can be effective in detecting subjects sensitively.

### Pupil dilation during visual search in AD patients

In control subjects, accumulating evidence indicates a link between pupil size and visual attention, in which the pupil diameter gets more enlarged as the cognitive load required for task performance increases (Gabay et al., 2011; Geng et al., 2015; Salvaggio et al., 2022). Pupil dilation with performance of the visual search task can be interpreted in terms of the cognitive demand required. Searching for a target from among 48 rings was a more cognitively demanding task than searching from among 4 rings, and required sustained and concentrated visual attention the task-related dilation of pupils during the visual search task was not as prominent as that of control subjects, which was observed in the 48 ring task but not in the 4 ring task (Figures 5A, B).

Pupil size modulation is important in the context of AD or even MCI, since task-related pupil dilation reflects the activity of the locus coeruleus (LC), which is one of the initial sites of subcortical tau deposition in early stage AD (Braak et al., 2011). According to Aston-Jones and Cohen (2005), LC neurons exhibit two modes of activity, phasic and tonic. Phasic LC activation is driven by the outcome of task-related decision processes and facilitates strategies for ensuing behaviors and to help optimize task performance (exploitation). Phasic LC activity regulates cortical encoding of salience information, which would be beneficial for the target detection (Vazey et al., 2018). When the utility of exploitation decreases, LC neurons exhibit a tonic activity mode, facilitating disengagement from the current task and switching to an alternative behavior (exploration).

In this context, modulation of pupillary responses is reported as a potential sensitive indicator for early risk for MCI and AD risk prediction, and even as a midlife indicator of risk for AD (Granholm et al., 2017; Kremen et al., 2019). This compensatory enhancement of task-related pupil dilation was observed in the context of a short-memory task such as the digit span. This task required more effort on the side of patients, and can be a response to not being able to memorize and recall properly, or even failed rehearsal of memorized digits during the memorization. Enhanced task-related pupil dilation may represent a compensatory effort to maintain performance through attentional engagement in the exploration mode, especially in amnestic MCI subjects; amnestic MCI patients may exert more effort in performing the task because they are closer to their maximum capacity for compensation (Riediger et al., 2006; Stern et al., 2018). In contrast, our task was performed more easily by most subjects participating and did not incur working memory or executive function, requiring less effort on the part of subjects. The task may be performed more ‘automatically’ or ‘subconsciously’ in the exploitation mode, and the task-related pupil dilation was diminished rather than enhanced in AD patients, which may reflect dysfunction of the LC. The modulation also showed a strong correlation with the MMSE score. Since the pupil size modulation in our task exhibited a high selectivity but low sensitivity, it can be useful for confirming diagnosis of dementia rather than screening of dementia in general public.

### Limitations of the study

There are some limitations in our study. First, the statistical power we obtained was relatively weak, which implies that we cannot apply our present experimental procedure directly to the clinical diagnosis of AD.

Second, the images we used in the visual scanning task are all line drawings, with black lines drawn on a white background (Figure 1A). Future research should adopt a more ecological or naturalistic design, with images simulating the outer world we see every day, which is much more complex, composed of more complex shapes and full of colors, containing many informative ROIs. For example, if we had used photographs or movies in the visual scanning task, the difference in the visual attention of AD patients and control subjects would be differentiated more clearly (Reading et al., 2012; Ramzaoui et al., 2018).

The third limitation is the small number of participants in this study, both AD patients and control subjects. We should increase the statistical power by studying more large-scale populations in the future.

The fourth limitation concerns the evaluation of pupil size change. In this study, we divided the pupil sizes by the averaged pupil sizes in the first trial of each subject, and this relative pupil size was analyzed. We took the pupil sizes in the first trial as a baseline because they are not affected by the execution of search tasks. Admittedly, the pupil size during the first trial may already be dilated by the cognitive effort and novelty effects, and may not necessarily be used as a reliable baseline. Unfortunately, we have not collected data for pupil size just-before the trials for a sufficient period. During the intersession period, the pupil size often deviated largely from the intrasession pupil size, partially because subjects moved their eyes during the period, and possibly because of distraction and/or mind wandering, i.e., the mental status was not stable during the intrasession period, so that it was difficult to take the intersession pupil size as baseline. As a compromise, we took the pupil sizes in the first trial, which was the period the subjects just started the session, as a baseline because they are not affected by the execution of search tasks. The same analysis was done both for AD patients and control subjects, which allowed comparison between the two groups.

## Conclusions

Most of the AD patients were able to complete the present task without much difficulty. This may be because the present task addressed attentional function related to visuospatial processing using the visual task and visual search in combination with eye tracking, instead of directly requiring responses to asked questions, which would necessitate explicit verbal processing and rely more on memory or executive function. Nevertheless, the task successfully visualized the multiple aspects of attention associated with cognitive decline, making it amenable even to AD patients with apathy and memory disturbance. The reduced fixation on informative ROIs in the visual memory task reflected impaired visuospatial attentional allocation due to defective executive/working memory function, leading to poorer recall of visual content, which could detect cognitive decline in AD patients with high sensitivity. Increased search time and saccade number until target detection in the visual search task indicated inefficient visual scanning, suggesting defective visual processing, especially in the serial search mode with a large number of items, which was related to top-down versus bottom-up attentional engagement. These results showed a high sensitivity in differentiating between AD patients and control subjects. On the other hand, decreased pupil size modulation during the visual search task suggested a diminished ability to increase visual attention to adapt to the cognitive load of the task and to optimize the performance, possibly associated with dysfunction of the LC. Pupil size modulation during the visual search task could be used to differentiate between subjects with normal cognition and those with cognitive decline because of its high specificity. Despite some limitations, considering the multiple aspects of visuospatial processing associated with attention in combination, we were able to detect aspects of early cognitive decline and this may become a new biomarker for the early detection and evaluation of cognitive decline that has high sensitivity and specificity.

## Data availability statement

The original contributions presented in this study are included in the article/supplementary material, further inquiries can be directed to the corresponding author.

## Ethics statement

The studies involving human participants were reviewed and approved by Ethics Committee at the University of Tokyo (approval no. 2411). The patients/participants provided their written informed consent to participate in this study.

## Author contributions

S-iT performed the experiments, collected the data, and prepared the figures. S-iT and YT wrote the main manuscript text and carried out patient recruitment. All authors reviewed the manuscript.
